# β-lapachone suppresses carcinogenesis of cervical cancer via interaction with AKT1

**DOI:** 10.3389/fphar.2025.1509568

**Published:** 2025-02-20

**Authors:** Pan Du, Yue Li, Anna Han, Mengying Wang, Jiajing Liu, Yingshi Piao, Liyan Chen

**Affiliations:** ^1^ Key Laboratory of Pathobiology (Yanbian University), State Ethnic Affairs Commission, Yanji, China; ^2^ Changchun Center for Disease Control and Prevention, Changchun, China; ^3^ Cancer Research Center, Yanbian University, Yanji, China

**Keywords:** β-lapachone, glucose metabolism, AKT1, EMT, cervical cancer

## Abstract

**Introduction:**

Cervical cancer is one of the most prevalent malignant tumors affecting women worldwide, and affected patients often face a poor prognosis due to its high drug resistance and recurrence rates. β-lapachone, a quinone compound originally extracted from natural plants, is an antitumor agent that specifically targets NQO1.

**Methods:**

CC cells were treated with varying concentrations of β-lapachone to examine its effects on glucose metabolism, proliferation, metastasis, angiogenesis, and EMT *in vitro*. The targets and action pathways of β-lapachone were identified using network pharmacology and molecular docking, with KEGG pathway enrichment analysis. Its effects and toxicity were verified *in vivo* using a nude mouse xenograft model.

**Results:**

β-lapachone significantly inhibited the proliferation and metastasis of cervical cancer cells by regulating glucose metabolism, reducing tumor angiogenesis, and suppressing epithelial-mesenchymal transition (EMT) in cells with high NQO1 expression. Furthermore, we identified the inactivation of the PI3K/AKT/mTOR pathway as the key mechanism underlying these effects. AKT1 was identified as a potential target of β-lapachone in modulating glucose metabolism and EMT in cervical cancer cells.

**Conclusion:**

These findings suggest that β-lapachone inhibits the malignant progression of cervical cancer by targeting AKT1 to regulate glucose metabolism in NQO1-overexpressing cells, providing a theoretical basis for developing novel therapeutic strategies for cervical cancer.

## Introduction

Cervical cancer (CC) remains one of the leading causes of cancer-related mortality among women worldwide, particularly in young and middle-aged populations (Siegel et al., 2024). Recent data indicate that by 2050, the World Health Organization (WHO) predicts CC will result in over 1 million deaths annually (Feng et al., 2019). While radiotherapy is a commonly used treatment, it often lacks specificity and is associated with significant side effects, leading to poor prognosis and high recurrence rates (Woo and Kim, 2022; Vaccarella et al., 2013). Therefore, it is crucial to identify novel targeted drugs with minimal side effects to treat CC.

β-lapachone, a 1,2-naphthoquinone compound first isolated from the lapacho tree (*Tabebuia avellanedae*) (Yu et al., 2014), has demonstrated diverse biological activities, including anti-inflammatory, antiviral, and antipsoriatic effects (Sanajou et al., 2021; Kee et al., 2017). In recent years, β-lapachone has gained attention for its selective antitumor activity in various cancers, including hepatocellular carcinoma, gastric carcinoma, and breast cancer (Zhou et al., 2021; Zheng et al., 2021a; Yang et al., 2017). Its effects are closely tied to NAD(P)H: quinone oxidoreductase 1 (NQO1), an enzyme that catalyzes the conversion of β-lapachone into unstable hydroquinone. This compound undergoes rapid autoxidation, producing large amounts of reactive oxygen species (ROS) that cause oxidative DNA damage. This, in turn, hyperactivates poly (ADP-ribose) polymerase-1 (PARP1), leading to depletion of NAD^+^/ATP and ultimately inducing cell death (Huang et al., 2016; Lamberti et al., 2018). Despite the established importance of β-lapachone–NQO1 interactions in various cancers, its therapeutic potential in CC remains unclear.

Reprogramming of glucose metabolism serves as a distinguishing feature of tumor cells, which provides sustained energy for tumor growth and metastasis (DeBerardinis et al., 2008; Dong et al., 2022). To achieve sustained proliferation, tumor cells usually adjust their metabolism and nutrient acquisition. Tumor cells are usually dependent on an environment in which aerobic glycolysis produces large amounts of ATP and lactate even when oxygen is sufficient. This cellular switch from the normal respiratory pathway to aerobic glycolysis is known as the Warburg effect (Wu et al., 2016; Gu et al., 2017; Christofk et al., 2008). Moreover, in addition to serving as an energy source for cancer cells, aerobic glycolysis promotes local tumor invasion and metastasis and facilitates tumor immune escape (Tamada et al., 2012; Kitamura et al., 2011). Therefore, the development of antitumor drugs targeting the glucose metabolism pathway has the potential to provide an effective approach to cancer treatment.

In this study, we demonstrated that β-lapachone exhibits significant antitumor effects against NQO1-overexpressing CC cells *in vitro* and *in vivo*. Mechanistic studies revealed that β-lapachone inhibits cell proliferation, metastasis, glucose metabolism, tumor angiogenesis, and EMT by targeting AKT1 to inactivate the PI3K/AKT/mTOR pathway.

## Materials and methods

### Chemical compounds and cell culture

β-lapachone was purchased from Medchem Express (HY-13555, United States) and was dissolved in dimethyl sulfoxide (DMSO, D8371, Solarbio, China) to prepare a stock solution of 10 mM and stored at −20°C. 740 Y-P was purchased from Medchem Express (HY-P0175, United States). Human cervical cancer cell lines SiHa, HeLa, C33a and HcerEpic were provided by the Oncology Research Center of Yanbian University, China. HeLa, C33a, HcerEpic, and SiHa cells were cultured in Dulbecco’s modified Eagle Medium (DMEM) supplemented with 1% penicillin-streptomycin and 10% fetal bovine serum (FBS). The cells were kept at a steady temperature of 37°C and exposed to 5% CO_2_.

### MTT assay

Cells were inoculated in 96-well plates and treated with different concentrations of β-lapachone for 24, 48 and 72 h. Subsequently, 100 μL of MTT (1 μg/mL) was introduced to each well and left to incubate away from light for 4 h. Finally, 100 μL of DMSO was introduced to each well, and the cells were incubated away from light for 10 min. The absorbance measurement at 490 nm was used to determine the OD value.

### Colony-formation assay

The cells were placed in 6-well plates at a concentration of 1,000 cells per well for the purpose of colony formation analysis. Subsequent to subjecting the cells to diverse concentrations of β-lapachone for a duration of 24 h on the subsequent day, the culture was sustained for a period of 10–14 days by substituting the drug-free DMEM medium. After the culture was finished, the cells were put in a 4% paraformaldehyde solution for 15 min, while Giemsa was stained for 30 min. Ultimately, a count of all the colonies was made, and pictures were acquired. We conducted three replications of each experiment.

### Wound healing assay

After seeding the cells into 6-well plates, we scratched the middle of each well with a 200 µL pipette tip the next day, once the cells had fully fused. Following PBS washing and incubation in DMEM medium containing varying β-lapachone concentrations, measurements of the wound’s distance were made and photos were taken at predetermined intervals.

### Transwell invasion and migration assays

For migration assay, the cells were routinely digested to prepare a cell suspension, and a serum-free DMEM culture with a cell density of 1 × 10^5^ was added to 200 µL in the upper chamber. The corresponding lower section was incubated with 800 µL of DMEM medium containing 10% FBS in a 37°C, 5% CO_2_ incubator. On the following day, the serum-free DMEM medium containing different concentrations of β-lapachone was added to the upper chamber, the liquid in the lower chamber was discarded, and 800 µL of DMEM medium containing 10% FBS was re-added to continue the culture. The upper chamber was fixed with cold 4% paraformaldehyde at room temperature for 20 min, followed by staining with hematoxylin for 10 min. Remove the non-migrating cells from the bottom of the upper chamber with a cotton swab. The stained cells were observed under a light microscope and processed for blocking, and the number of stained cells was measured and analyzed using ImageJ software. In the invasion assay, the bottom of the transwell was coated with matrigel (matrigel and DMEM culture solution in a 1:6 ratio) 1 day in advance. The remaining experimental procedures were the same as for the migration experiments.

### ATP, lactate and glucosen level assays

Cells were plated in 6-well plates and then treated with different doses of β-lapafenone for 24 h. The cells and supernatants were collected separately, and then ATP, lactate, and glucose levels were measured according to the requirements related to the Glucose assay kit (Applygen, E1011), ATP assay kit and Lactate assay kit (Njjcbio, A095/A019-2, China).

### Tube formation assay

The 96-well plate was pre-chilled with 25 µL of matrigel in a 1:1 ratio and incubated at 37°C. Siha and C33a cells were treated with different concentrations of β-lapachone, and the supernatant was extracted and set aside after 6 h of incubation. Prepare HUVEC cell suspension with a density of 3 × 10^4^, 50 µL/well. The extracted 150 µL of supernatant and HUVEC cell suspension were mixed and added to a 96-well plate and incubated at 37°C in a 5% CO_2_ incubator. The tube formation was imaged at 30 min intervals with a microscope (BX71, Olympus, Japan), and the resulting pictures were evaluated using ImageJ’s Angiogenesis Analyzer plugin.

### Plasmids and transfection

The siRNA molecules targeting AKT1 were synthesized, purified and purchased by Hippobio (zhejiang, China). All plasmids were transfected with Lipofectamine™ 3000 transfection reagent (#L3000015, ThermoFisher, shanghai, China) following the manufacturer’s instructions. The siRNA sequences used were as follows: siAKT1#1: 5′-GGA CAA GGA CGG GCA CAU UAA TT-3’; siAKT1#2: 5′-CUA UGG CGC UGA GAU UGU GUC TT-3’; siAKT1#3: 5′-CGC CUC ACC AUG AAC GAG UUU TT-3’.

### Western blot analysis

Cells were treated with different concentrations of β-lapachone for 24 h. The RIPA lysate, protease inhibitor, and phosphatase inhibitor were combined with the entire cell lysate in a ratio of 100:1:1, and the BCA kit (Beyotime, China) was used to measure the protein concentration. Through the utilization of sodium dodecyl sulfate-polyacrylamide gel electrophoresis (SDS-PAGE), an equal proportion of the overall 40 μg protein was extracted, isolated, and subsequently transferred onto PVDF membranes (Millipore, United States). Following 1 h of blocking with 5% skimmed milk, a primary antibody was subjected to an overnight incubation at 4°C with the PVDF membrane. The primary antibodies used in this study were as follows: anti-AKT (CST, #4060, 1:1000), anti-E-cadherin (Proteintech, #20874, 1:1000), anti-G6PC (Proteintech, #22169, 1:1000), anti-GADPH (CST, #5174, 1:1000), anti-mTOR (CST, #2983, 1:1000), anti-NQO1 (CST, #3187, 1:1000), anti-PKM2 (Proteintech, #15822, 1:1000), anti-Snail (Proteintech, #13099, 1:1000), anti-Vimentin (Proteintech, #10366, 1:1000), anti-VEGF (Proteintech, #19003, 1:1000), anti-β-actin (Cwbio, #CW0264, 1:1000). The membranes were treated with a secondary antibody for one hour at room temperature the following day after being rinsed with TBST. Protein bands were examined using electrochemiluminescence (ECL) imaging equipment (Amersham imager 600, United States), and the bands were analyzed using Image Lab software.

### Xenegraft model

The Experimental Animal Center of Yanbian University provided us with female BALB/C nude mice that were 4 weeks old. The Animal Research Ethics Committee granted approval to all experiments, and all creatures were kept in a sterile atmosphere (Registration Number: YD20231120001; Approval Date: 20 November 2023). For the *in vivo* xenograft model, SiHa cells (5 × 10^6^) and matrigel were combined proportionately and subcutaneously into the lower dorsal side of naked mice. Following the injection, the mice were allocated into two sets of six in a random manner. During the intraperitoneal injection of β-lapachone (5 mg/kg) every other day for a duration of 14 days, measurements were taken for body weight and tumor size. After the experiment, the mice were euthanized, all tumor samples were collected, recorded, and further examined.

### Rescue experiments

In accordance with the instructions provided by the manufacturer, the PI3K activator 740 Y-P was dissolved in DMSO until it reached a final concentration of 5 μM. Siha cells underwent a 24 h treatment with β-lapachone at a concentration of 4 μM, while C33a cells were subjected to a 24 h treatment at a concentration of 6 μM prior to receiving 740 Y-P for the same duration. A western blot was employed to ascertain the expression of AKT, p-AKT, mTOR, p-mTOR, and EMT-related proteins.

### Online database extraction

The GEPIA online database (http://gepia.cancer-pku.cn/) was utilized to analyze the expression of NQO1 in cervical cancer patients (Tang et al., 2017). Through the utilization of the websites Pharm Mapper and Gene Cards, it is possible to ascertain the target genes for β-lapachone in cervical cancer (https://www.lilab-ecust.cn/pharmmapper/, https://www. genecards. org/). Gene Ontology enrichment and Kyoto Encyclopedia of Genes and Genomes (KEGG) pathway analyses were conducted on the target genes for β-lapachone using the KOBAS tool (Bu et al., 2021).

### Molecular docking

The 3D structure of AKT1 protein (PDB ID:1UNQ) was first obtained by searching from the Research Collaboratory for Structural Bioinformatics Protein Data Bank (RCSB PDB, http://www.rcsb.org/pdb/). The SDF format of β-lapachone was obtained from pubchem database (https://pubchem.ncbi.nlm.nih.gov) and converted to PDB format by Open Babel. Proteins were dehydrogenated, hydrogenated to calculate charge and converted to pdbqt format using AutoDocktools 1.5.7 software, ligands were hydrogenated, torsional forces were determined and converted to pdbqt format. Docking box coordinates were determined and molecular docking operations were performed using Autodock vina software. And PyMOL 2.1.0 was used to visualize the presentation and obtain the 3D analytical maps; Discovery studio software was used to visualize the interactions between the tested compounds and key residues.

### Statistical analysis

Data from the experiment were statistically analyzed using Graphpad Prism 10.0. The standard deviation is used to ± express the data as means. The data was compared among different groups using either a one-way ANOVA or a paired student t-test. All results were performed in at least three independent experiments. *P* < 0.05 showed that the difference was statistically significant.

## Results

### β-lapachone inhibits the proliferation of cervical cancer cells with high NQO1 expression

**FIGURE 1:**
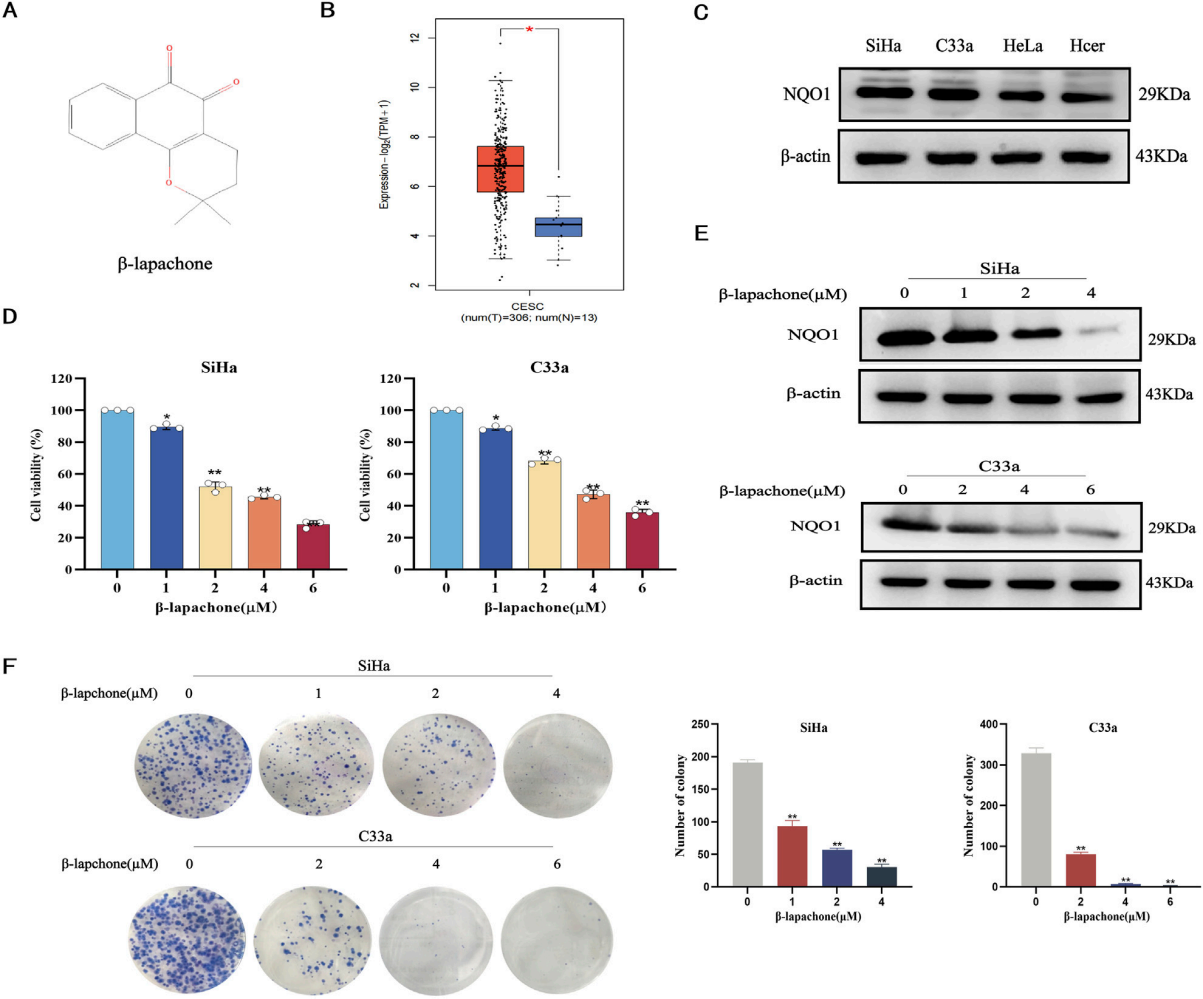
β-Lapachone inhibits the proliferation of cervical cancer cells. **(A)** Chemical structures of β-lapachone. **(B)** Expression of NQO1 in cervical cancer and normal tissues were analyzed by GEPIA database. **(C)** NQO1 protein levels in cancer cell lines and normal cells were detected by Western blot. **(D)** Cell viability was detected by MTT assay after treatment of cervical cancer cells with different concentrations of β-lapachone. **(E)** The expression of NQO1 protein in SiHa and C33a cells treated with β-lapachone for 24 h. **(F)** The effect of β-lapachone on cell proliferation was detected by colony formation assay. Error bars represent the means ± SD of three independent experiments. *P < 0.05, **P < 0.01 vs. 0 μM β-lapachone group.

To investigate the effects of β-lapachone (Figure 1A) on CC, we first analyzed NQO1 expression in CC tissues using the GEPIA online database. The results indicated that NQO1 expression is significantly higher in CC tissues compared to normal tissues (Figure 1B). Western blotting further confirmed elevated NQO1 levels in cervical CC lines (SiHa and C33a) compared to normal cervical epithelial cells (HcerEpic). Among these, SiHa and C33a cells, which exhibited the highest NQO1 expression, were selected for subsequent experiments in which they were treated with varying concentrations of β-lapachone (Figure 1C). MTT assays revealed that β-lapachone significantly inhibited the survival of SiHa and C33a cells (Figure 1D). Then, we screened the optimal concentration of β-lapachone for drug action on CC cells, revealing that treatment with β-lapachone also reduced NQO1 expression levels in these cells (Figure 1E). Furthermore, colony formation assays demonstrated that β-lapachone dose-dependently suppressed the colony-forming ability of CC cells compared to the control group (Figure 1F). Collectively, these findings highlight the inhibitory effects of β-lapachone on CC cell proliferation.

### β-lapachone attenuates cervical cancer cell migration, invasion, EMT, and angiogenesis

**FIGURE 2:**
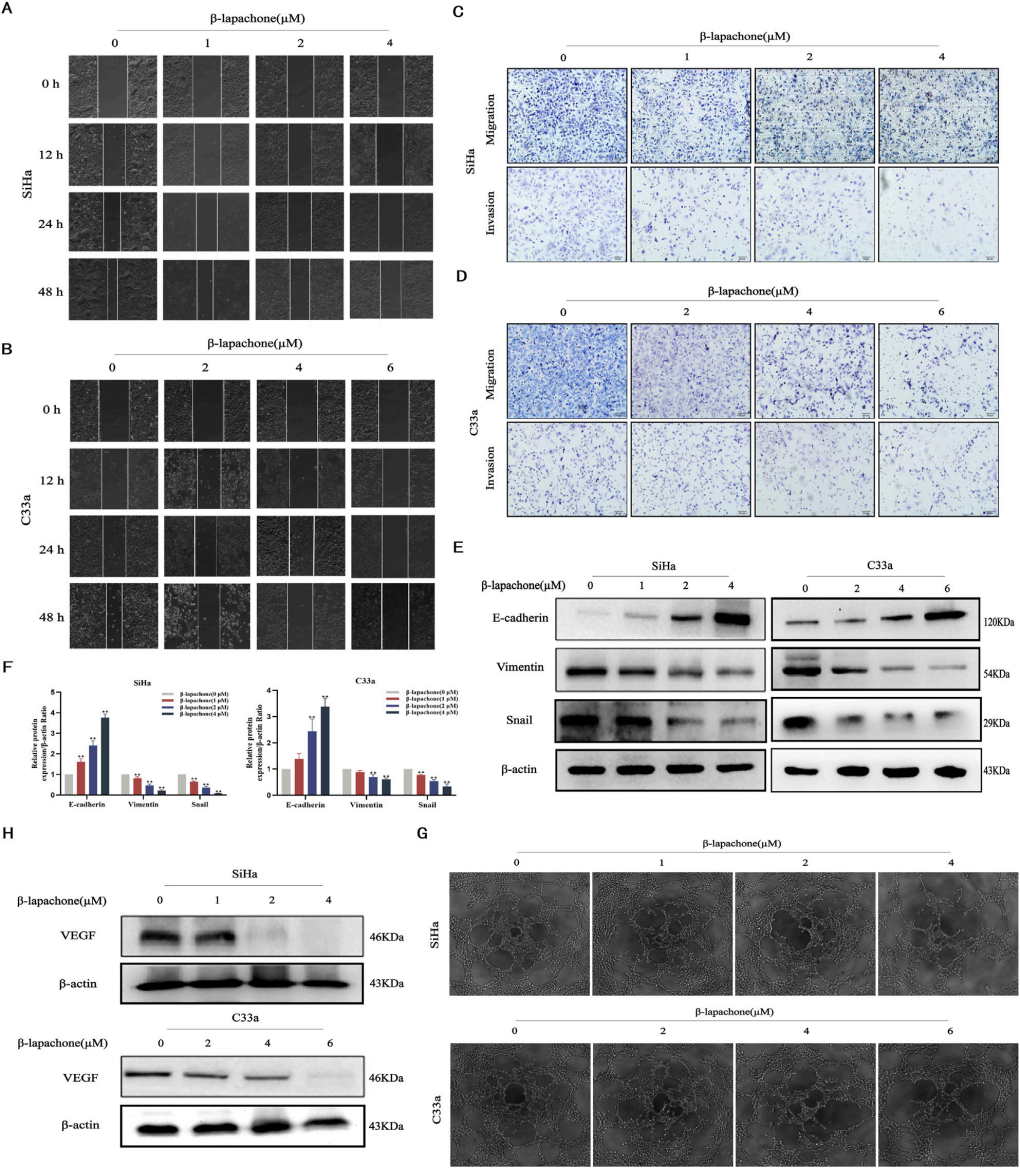
β-Lapachone attenuated cervical cancer cell migration, invasion, EMT and angiogenesis. **(A, B)** Wound healing assay was used to reflect the migration ability of SiHa and C33a cells after treatment with the indicated concentrations (0–6 µM) of β-lapachone. **(C, D)** Transwell assay was used to detect the effect of the indicated concentrations of β-lapachone treatment on the migration and invasion ability of CC cells. **(E, F)** Cells were treated with β-lapachone (0–6 µM) for 24 h, and protein levels of E-cadherin, Vimentin, and Snail were analyzed by Western blot. **(G)** The supernatant from cells treated with β-lapachone (0–6 μM) for 24 h was collected for the tube formation assay. **(H)** After treating cells with β-lapachone (0–6 µM), VEGF protein expression was detected by Western blot. Error bars represent the means ± SD of three independent experiments. *P < 0.05, **P < 0.01 vs. 0 μM β-lapachone group.

Tumor cell proliferation and metastasis are critical processes in cancer progression. We evaluated the effects of β-lapachone on CC cell migration and invasion using wound healing and Transwell assays. Wound healing assays showed that β-lapachone significantly reduced the wound closure rates for SiHa and C33a cells after 12 h (Figures 2A, B). Similarly, Transwell assays assessing longitudinal migration revealed that β-lapachone markedly decreased the number of migratory cells (Figures 2C, D). Consistent with this, β-lapachone treatment led to a notable reduction in the quantity of invasive cells. Therefore, these findings suggest that β-lapachone can attenuate the migration and invasion of CC cells.

The epithelial-mesenchymal transition (EMT) is essential for the development of tumors. To delve deeper into the potential involvement of the EMT process in the impact of β-lapachone on CC cells, we analyzed the expression of EMT-related markers via Western blotting. The results showed that the expression level of the epithelial marker E-cadherin was significantly elevated in CC cells after β-lapachone treatment. In contrast, there was a marked decrease in the expression of the mesenchymal markers Vimentin and Snail (Figures 2E, F). To further elucidate the mechanisms by which β-lapachone inhibits CC metastasis, supernatants from β-lapachone-treated CC cells were collected and used to culture HUVECs. Microscopic observation of tube formation assay results revealed that β-lapachone significantly inhibited the angiogenic capacity of HUVECs in a concentration-dependent manner (Figure 2G). Additionally, Western blotting showed that β-lapachone substantially suppressed the expression of the angiogenesis-related protein VEGF in treated cells (Figure 2H). These findings collectively suggest that β-lapachone inhibits CC metastasis and progression by suppressing angiogenesis and EMT induction.

### β-lapachone regulates glucose metabolism reprogramming in cervical cancer cells

**FIGURE 3:**
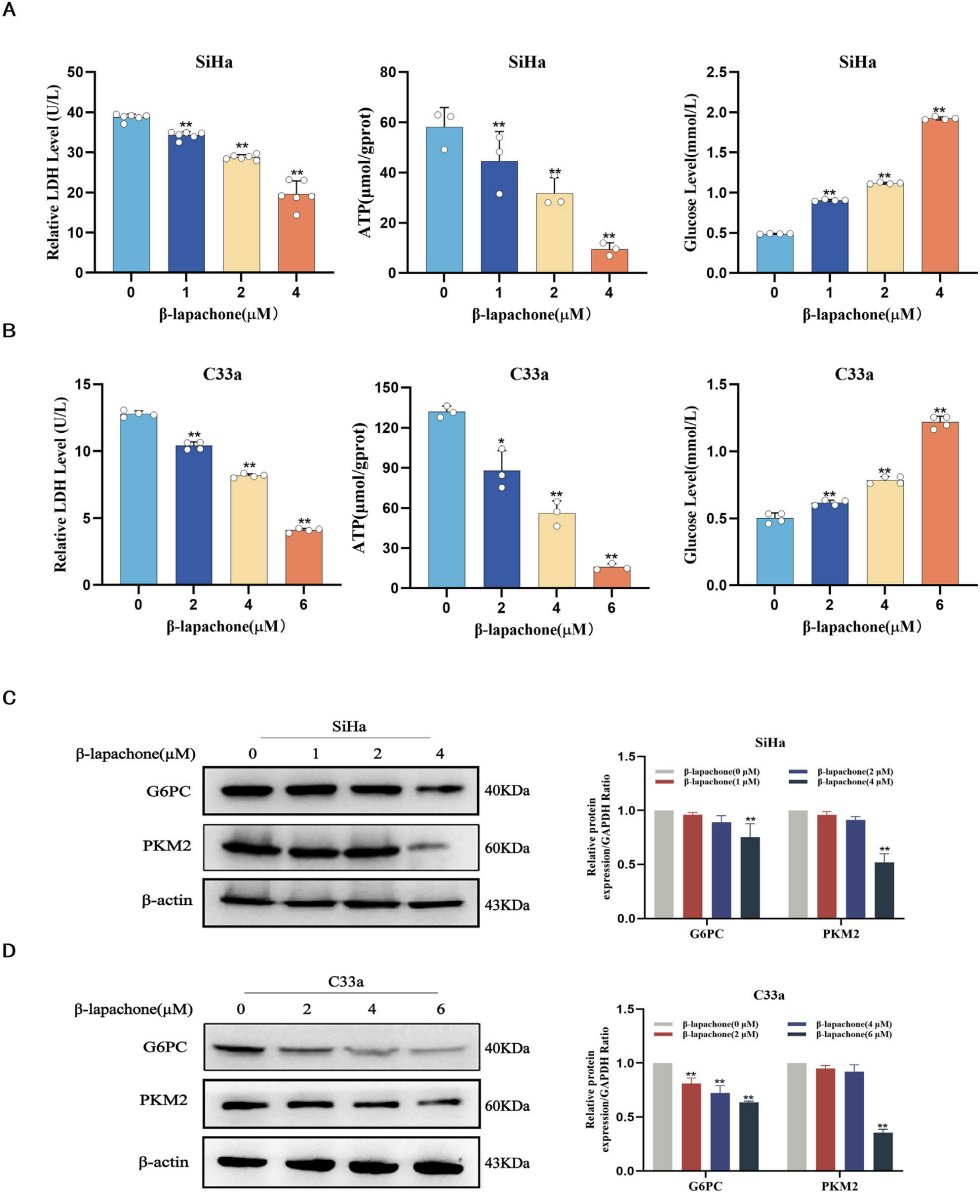
β-lapachone regulates glucose metabolism reprogramming in cervical cancer cells. **(A, B)** SiHa and C33a cells were treated with the indicated concentrations of β-lapachone (0–6 µM) for 24 h. Relative levels of ATP, Lactate and Glucose were determined. **(C, D)** After treating cells with β-lapachone (0–6 µM), PKM2 and G6PC protein expression was detected by Western blot. Error bars represent the means ± SD of three independent experiments. *P < 0.05, **P < 0.01 vs. 0 μM β-lapachone group.

Metabolic reprogramming is a hallmark of cancer, sustaining tumor cell growth, metastasis, survival, and treatment resistance. To evaluate the effect of β-lapachone on glucose metabolism in CC cells, we measured ATP, lactate, and glucose levels after treatment. The results showed a significant increase in intracellular glucose levels and a reduction in ATP and lactate dehydrogenase (LDH) levels following β-lapachone treatment (Figures 3A, B). To further investigate the molecular mechanisms underlying the effects of β-lapachone, we analyzed the expression of the glucose metabolism-associated proteins G6PC and PKM2 in CC cells. Western blotting revealed a significant reduction in G6PC and PKM2 expression levels in β-lapachone-treated SiHa and C33a cells, with more pronounced effects observed in C33a cells (Figures 3C, D). These findings suggest that β-lapachone disrupts glucose metabolism, thereby preventing CC progression.

### β-lapachone inhibits cervical cancer progression and metastasis via the PI3K/AKT/mTOR signaling pathway

**FIGURE 4:**
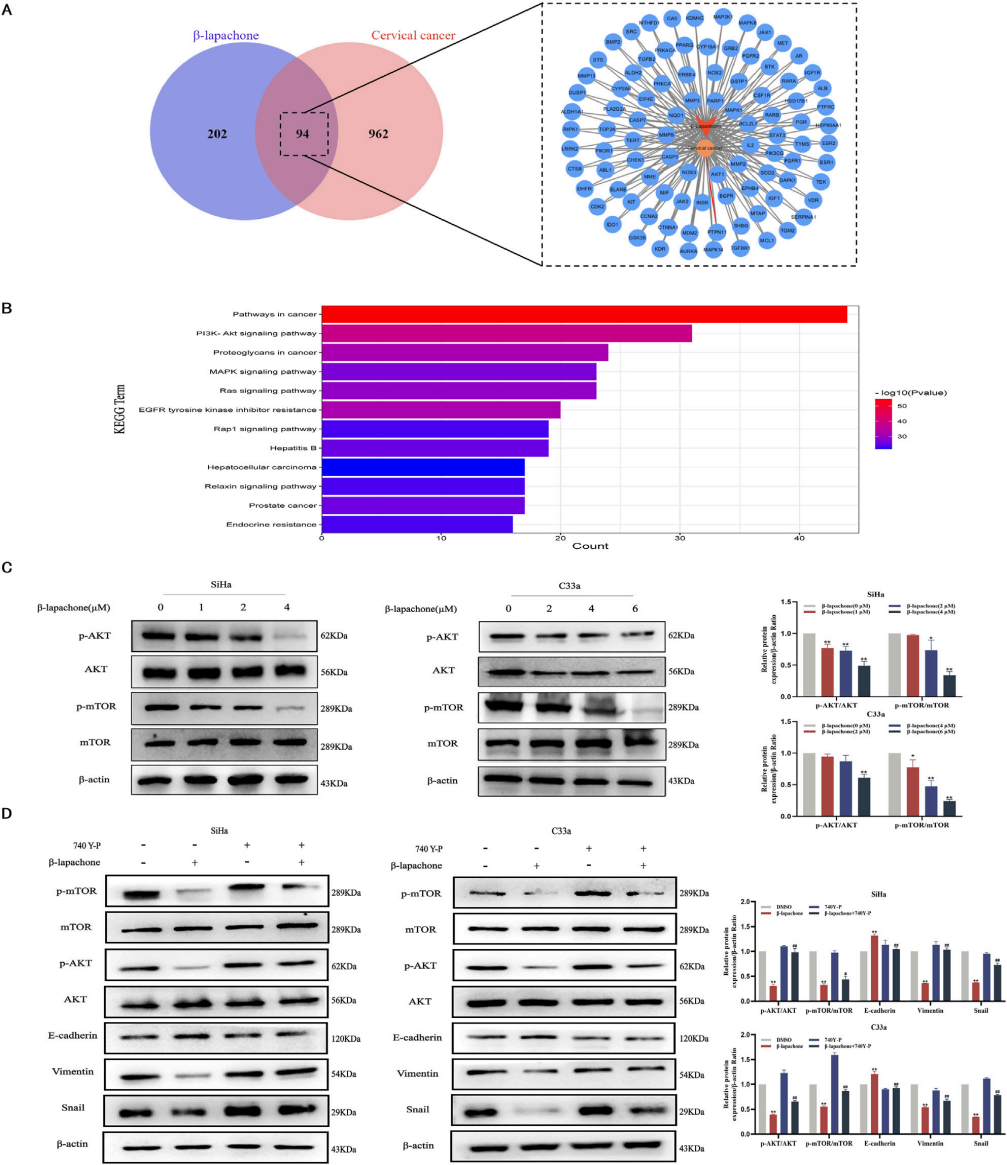
β-lapachone inhibits cervical cancer progression and metastasis via the PI3K/AKT/mTOR signaling pathway. **(A)** Prediction of β-lapachone related target genes in cervical cancer. **(B)** KEGG pathway enrichment analysis. **(C)** After treating cells with β-lapachone (0–6 µM), p-AKT/t-AKT, p-mTOR/t-mTOR protein expression was detected by Western blot. **(D)** The expression of proteins related to the PI3K/AKT/mTOR pathway and EMT process in CC cells was detected by Western blot after treating with β-lapachone and the pathway activator 740 Y-P. Error bars represent the means ± SD of three independent experiments. *P < 0.05, **P < 0.01 vs. DMSO group, #*P* < 0.05, ##*P* < 0.01 vs. β-lapachone group.

Using the GeneCards and PharmMapper databases, we identified 94 potential common targets of β-lapachone and CC (Figure 4A). KEGG pathway enrichment analysis of these targets suggested that the PI3K/AKT/mTOR signaling pathway plays a pivotal role in the inhibitory effects of β-lapachone on CC (Figure 4B). To validate this prediction, we analyzed the expression levels of proteins related to the PI3K/AKT/mTOR signaling pathway in β-lapachone-treated CC cells. Western blotting revealed a significant reduction in p-mTOR/mTOR and p-AKT/AKT levels following β-lapachone treatment (Figure 4C).

To confirm the involvement of the PI3K/AKT/mTOR pathway in the tumor-suppressive effects of β-lapachone, we conducted rescue experiments using the PI3K pathway activator 740Y-P. Treatment with 740Y-P reversed the β-lapachone-induced suppression of p-AKT and p-mTOR expression. Additionally, 740Y-P restored β-lapachone-induced changes in EMT markers, including E-cadherin, Snail, and Vimentin (Figure 4D). Collectively, these results indicate that β-lapachone suppresses CC progression and metastasis by inactivating the PI3K/AKT/mTOR signaling pathway.

### β-lapachone inhibits glucose metabolism and EMT induction in cervical cancer cells by targeting AKT1

**FIGURE 5:**
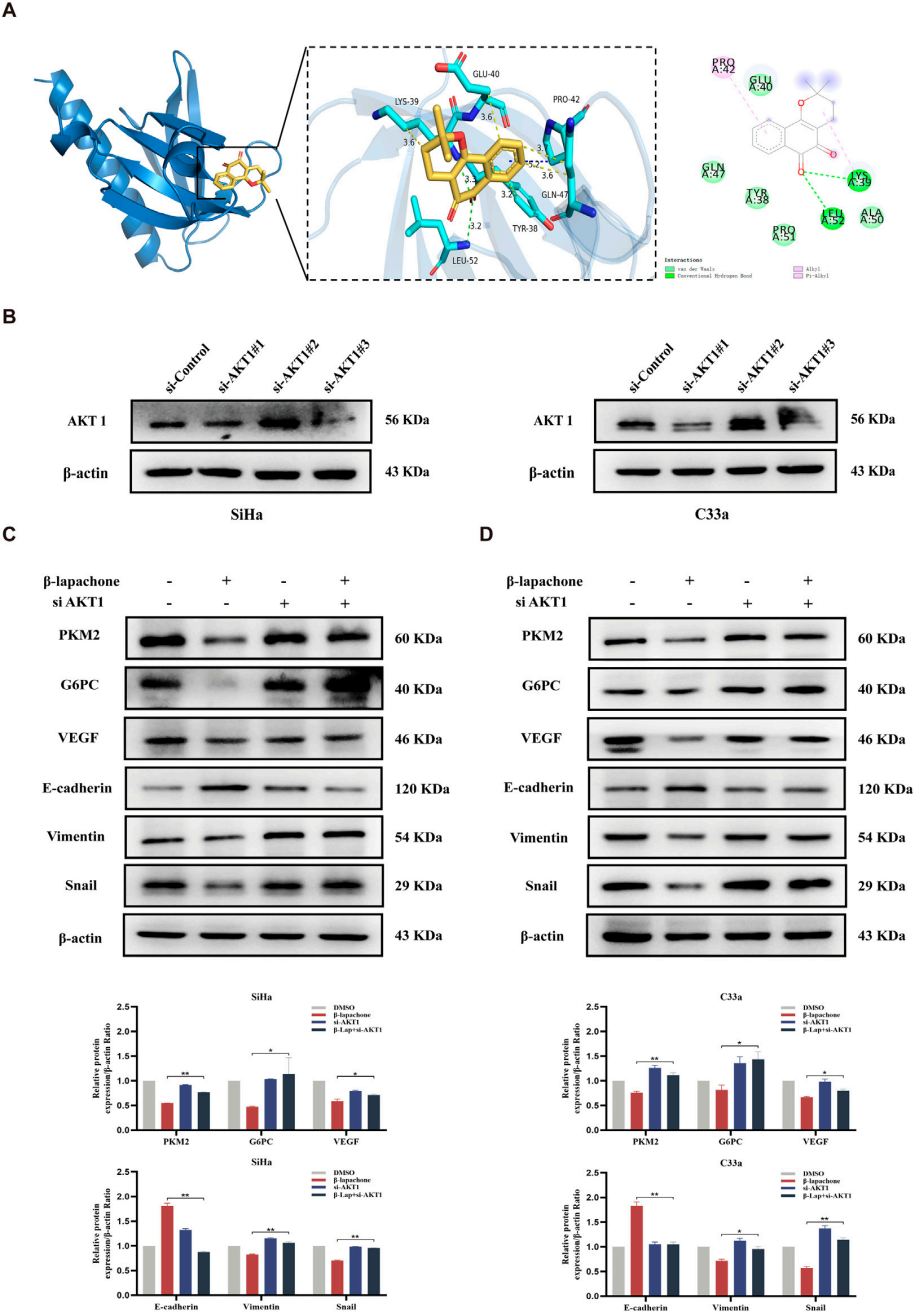
β-lapachone inhibits glucose metabolism and EMT process in CC cells by targeting AKT1. **(A)** Docking analysis for predicting the binding mode of β-lapachone to AKT1. **(B)** Western blot analysis of AKT1 in CC cells transfected with siRNA-control, siRNA-AKT1-1、siRNA-AKT1-2 and siRNA-AKT1-3. **(C, D)** CC cells with AKT1 knockdown were treated with β-lapachone for 24 h, and protein expression levels were analyzed by Western blot. *P < 0.05, **P < 0.01 vs. β-lapachone group.

To identify the specific candidate target proteins of β-lapachone involved in inhibiting CC progression, we combined target prediction results with pathway analysis and hypothesized that AKT1, a key protein in the PI3K/AKT/mTOR pathway, is involved in β-lapachone’s regulation of CC cells. Molecular docking using AutoDock Vina revealed strong binding between β-lapachone and AKT1, with a binding free energy of −6.7 kcal/mol and the formation of two hydrogen bonds at residues LYS39 and LEU52 (Figure 5A). To validate the role of AKT1, we knocked down AKT1 expression in CC cells (Figure 5B). AKT1 knockdown significantly attenuated the inhibitory effects of β-lapachone on the glucose metabolism markers PKM2 and G6PC. Similarly, the regulation of the EMT-related proteins E-cadherin, Snail, and Vimentin by β-lapachone was also abrogated by AKT1 knockdown (Figures 5C, D). These findings thus suggest that β-lapachone inhibits glucose metabolism and the EMT process in CC cells by targeting AKT1.

### β-lapachone inhibits the progression of cervical cancer *in vivo*

**FIGURE 6:**
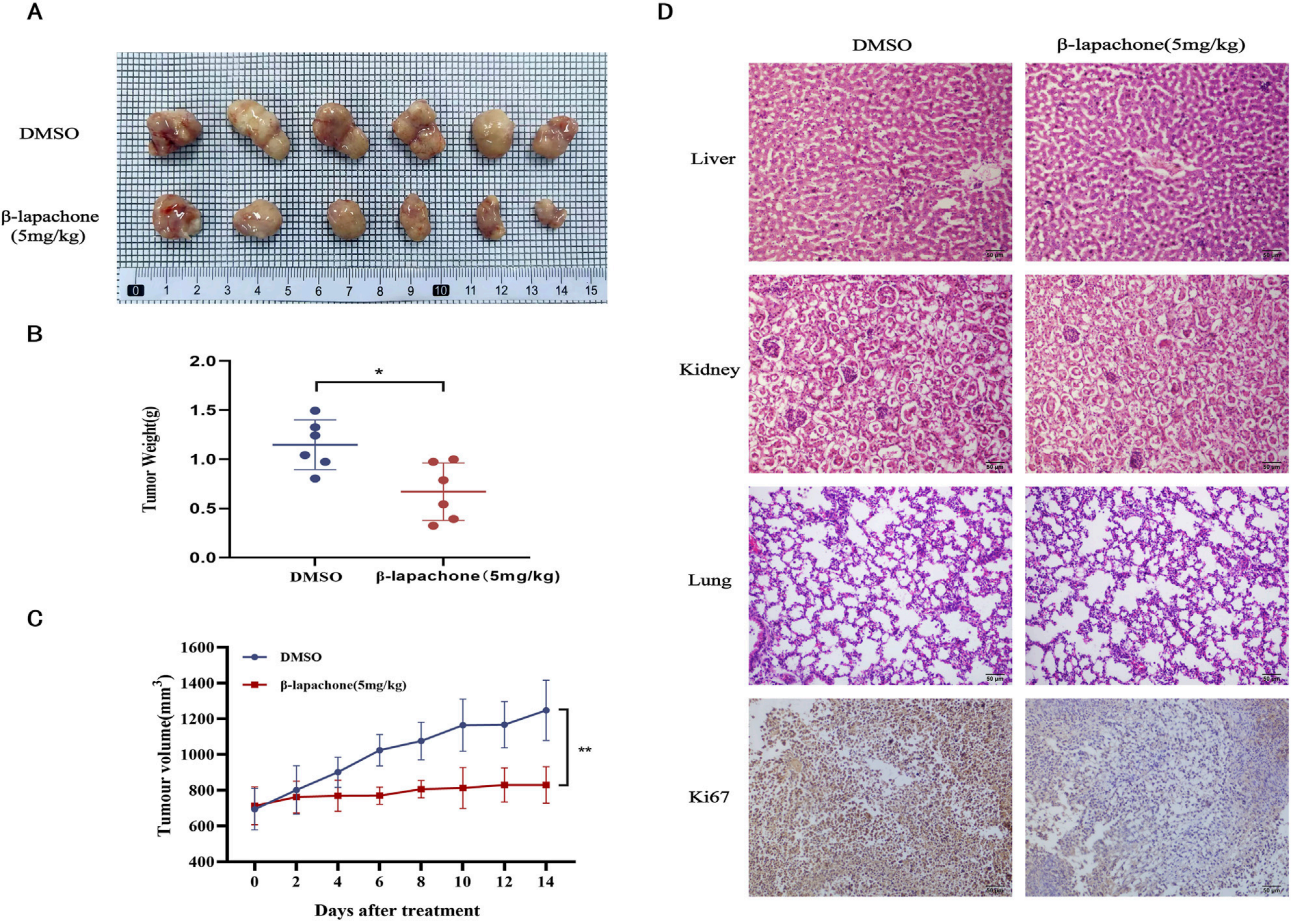
β-Lapachone inhibits tumor growth *in vivo*. **(A)** Images of subcutaneous xenograft tumors in the treatment group β-lapachone and control group. **(B)** Weight statistics of subcutaneous xenograft tumors in the treatment and control groups. **(C)** Growth volume statistics of subcutaneous xenograft tumors in the treatment and control groups during treatment with β-lapachone. **(D)** Representative immunohistochemical staining images of Ki67 expression in grafted tumor tissues from various groups of mice; Representative H&E stained images of liver, kidney and lung tissues from each group of mice. Scale bar: 50 μm *P < 0.05, **P < 0.01.

Finally, a nude mouse xenograft tumor model was established to examine the inhibitory effect of β-lapachone on CC *in vivo*. β-lapachone (5 mg/kg) was injected intraperitoneally every other day. The mice were euthanized 2 weeks after initiating treatment. Compared with those in the control group, the tumor weights and volumes in the treatment groups were significantly reduced (Figures 6A–C). In addition, no morphological changes were observed in the kidneys, lungs, and livers of mice in the β-lapachone treatment group. Immunohistochemical analysis of the tumor tissues from these nude mice revealed that the expression of the proliferation-associated antigen Ki67 was reduced in the β-lapachone-treated group relative to the control group (Figure 6D). Together, these findings demonstrate that β-lapachone exhibits a substantial inhibitory effect on the proliferation of CC tumors *in vivo* with no obvious toxicity.

## Discussion

β-lapachone, as a natural product, has attracted attention for its therapeutic potential in a variety of tumors (Gomes et al., 2021; Gong et al., 2021). Recent studies have shown that β-lapachone exerts its antitumor effects by inducing NQO1-mediated redox cycling, forming peroxides that damage DNA and lead to the overactivation of PARP, which in turn leads to ATP depletion. Tumors with high NQO1 expression, such as hepatocellular carcinoma, pancreatic cancer, and breast cancer, are thus particularly sensitive to β-lapachone (Yang et al., 2017; Li et al., 2011; Zhao et al., 2021; Qadir et al., 2022). In this study, we explored the potential anti-tumor effects of β-lapachone in CC *in vitro* and *in vivo*. The GEPIA database analysis revealed that NQO1 is highly expressed in CC, a finding further confirmed by the significantly higher expression of NQO1 in CC cells compared to normal cervical epithelial cells. Further investigation demonstrated that β-lapachone effectively inhibited CC cells with high NQO1 expression, exhibiting selective targeting properties. Additionally, β-lapachone showed low toxicity to HcerEpic cells (Supplementary Figure S1). Significant reductions in HcerEpic cell viability were observed only at high β-lapachone concentrations, highlighting the importance of selecting appropriate therapeutic doses to maximize anticancer efficacy. β-lapachone treatment resulted in a dose-dependent reduction in CC cell viability and colony-forming ability, particularly in cells with higher levels of NQO1 expression. Consistent with these findings, a xenograft model demonstrated that β-lapachone significantly reduced tumor weight and volume without apparent toxicity, aligning with the *in vitro* results. We hypothesize that the primarily cytostatic, rather than cytotoxic, effects of β-lapachone may stem from differences in NQO1 expression between normal and tumor tissues and that β-lapachone exerts its oncostatic effects mainly through the generation of peroxides via NQO1-mediated redox cycling, which then affects the DNA damage repair mechanism rather than inducing cell death directly.

Aberrant cellular metabolism is a hallmark of cancer, with tumor cells requiring large amounts of glucose to produce lactic acid via aerobic glycolysis. This unique metabolic process supports cancer cell energy needs and promotes survival (Huang et al., 2021; Bose and Le, 2018). Research has shown increased glycogen levels in various cancers, such as breast and ovarian cancers, with glycogen levels being inversely correlated with replication rates (Yang et al., 2016). In our study, we observed significantly elevated glucose levels in CC cells treated with β-lapachone, accompanied by marked reductions in ATP and lactate levels. These findings suggest that β-lapachone disrupts the aerobic glycolytic pathway critical for CC cell survival. PKM2, a key enzyme in the Warburg effect, catalyzes the final stage of aerobic glycolysis and often exists as a low-activity dimer in tumor cells (Sun et al., 2011; Liu et al., 2017). PKM2 facilitates cancer cell growth by enhancing macromolecular synthesis through the pentose phosphate pathway (Zhu et al., 2022). In addition, G6PC, a key enzyme in glucose homeostasis, plays a crucial role in gluconeogenesis and glycogenolysis, with its inhibition significantly slowing CC growth (Zhu et al., 2021; Singh et al., 2018). β-lapachone produces ROS, which are converted to hydrogen peroxide, leading to excessive DNA damage (Ross and Siegel, 2017; Hong et al., 2019). This overactivates PARP1, resulting in the depletion of NAD^+^ and ATP, ultimately impairing glycolysis, redox balance, and downstream metabolic processes (Silvers et al., 2017). Previous studies have also shown that β-lapachone regulates glucose metabolism in tumor types such as lung, pancreatic, and colorectal cancers (Huang et al., 2016; Mahar et al., 2021). Consistent with these findings, we observed the downregulation of PKM2 and G6PC expression in CC cells treated with β-lapachone. Collectively, our results confirm that β-lapachone reduces ATP and LDH levels by modulating PKM2 and G6PC expression, thereby preventing CC progression.

EMT induction is a biological process wherein epithelial cells lose their characteristics and acquire mesenchymal traits, enhancing metastatic potential and promoting cancer progression (Li et al., 2019; Hisano and Hla, 2019). Western blotting in this study demonstrated that β-lapachone downregulated mesenchymal markers Vimentin and Snail while upregulating the epithelial marker E-cadherin. This suggests that β-lapachone inhibits CC cell migration and invasion by suppressing EMT activity. Angiogenesis, another critical factor in tumor growth, facilitates metastasis by promoting endothelial cell proliferation and new tumor vessel formation (Lugano et al., 2020; Plate et al., 2012; Shibuya, 2011). VEGF and its receptor VEGFR play a pivotal role in angiogenesis, making them key targets for anti-angiogenic cancer therapies (Sharma et al., 2017). Consistent with these observations, our study revealed that β-lapachone significantly suppressed VEGF protein expression in CC cells, reducing microtubule formation in HUVECs. These findings suggest that β-lapachone inhibits CC progression by targeting both angiogenesis and EMT induction.

The PI3K/AKT/mTOR signaling pathway regulates cell proliferation, survival, and metabolism, with its dysregulation frequently observed in tumors (Reddy et al., 2020; Zheng et al., 2021b). This pathway is an attractive therapeutic target in various cancers, including CC (Yang et al., 2019). In this study, KEGG pathway enrichment analysis indicated that β-lapachone exerts its effects on CC cells through the PI3K/AKT/mTOR pathway. We verified the predicted results via Western blotting, revealing a significant reduction in p-AKT and p-mTOR levels in CC cells. Subsequently, we performed rescue experiments using the pathway activator 740Y-P and found that the expression of proteins related to the PI3K/AKT/mTOR signaling pathway and EMT process was significantly reversed by β-lapachone treatment in CC cells. More importantly, network pharmacology and molecular docking studies identified AKT1 as a potential β-lapachone target, and AKT1 knockdown significantly attenuated the inhibitory effects of β-lapachone on glucose metabolism and EMT in CC cells. These findings suggest that β-lapachone inhibits CC progression by targeting the PI3K/AKT/mTOR signaling pathway.

**FIGURE 7:**
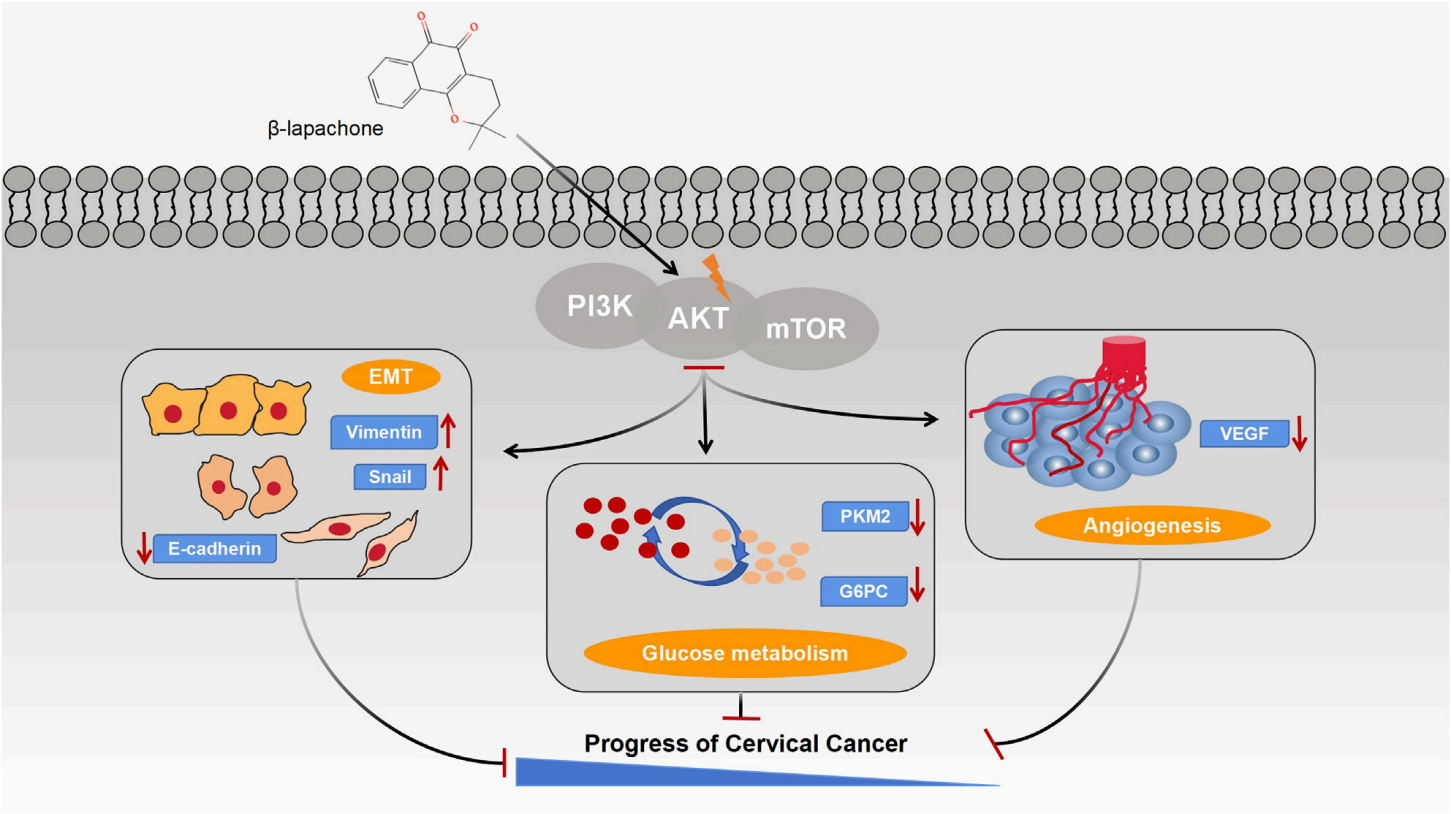
Schematic illustration of the mechanisms by which β-lapachone regulates glucose metabolism, EMT process and angiogenesis in cervical cancer through the PI3K/AKT/mTOR signaling pathway.

In conclusion, our study demonstrates that β-lapachone, an antitumor therapeutic agent targeting high NQO1 expression, modulates glucose metabolism, suppresses angiogenesis, and inhibits the EMT process by targeting AKT1. These effects collectively prevent the malignant progression of cervical cancer (Figure 7). Together, this work provides a theoretical foundation for developing β-lapachone as a therapeutic agent for cervical cancer.

## Data Availability

The original contributions presented in the study are included in the article/Supplementary Material, further inquiries can be directed to the corresponding author.

## References

[B1] Bose S. Le A. (2018). Glucose metabolism in cancer. Adv. Exp. Med. Biol. 1063, 3–12. 10.1007/978-3-319-77736-8_1 29946772

[B2] Bu D. Luo H. Huo P. Wang Z. Zhang S. He Z. (2021). KOBAS-i: intelligent prioritization and exploratory visualization of biological functions for gene enrichment analysis. Nucleic Acids Res. 49, W317–W325. 10.1093/nar/gkab447 34086934 PMC8265193

[B3] Christofk H. R. Vander Heiden M. G. Wu N. Asara J. M. Cantley L. C. (2008). Pyruvate kinase M2 is a phosphotyrosine-binding protein. Nature 452, 181–186. 10.1038/nature06667 18337815

[B4] DeBerardinis R. J. Lum J. J. Hatzivassiliou G. Thompson C. B. (2008). The biology of cancer: metabolic reprogramming fuels cell growth and proliferation. Cell Metab. 7, 11–20. 10.1016/j.cmet.2007.10.002 18177721

[B5] Dong S. Li W. Li X. Wang Z. Chen Z. Shi H. (2022). Glucose metabolism and tumour microenvironment in pancreatic cancer: a key link in cancer progression. Front. Immunol. 13, 1038650. 10.3389/fimmu.2022.1038650 36578477 PMC9792100

[B6] Feng R. M. Zong Y. N. Cao S. M. Xu R. H. (2019). Current cancer situation in China: good or bad news from the 2018 Global Cancer Statistics? Cancer Commun. (Lond) 39 (1), 22. 10.1186/s40880-019-0368-6 31030667 PMC6487510

[B7] Gomes C. L. de Albuquerque Wanderley Sales V. Gomes de Melo C. Ferreira da S. R. M. Vicente Nishimura R. H. Rolim L. A. (2021). Beta-lapachone: natural occurrence, physicochemical properties, biological activities, toxicity and synthesis. Phytochemistry 186, 112713. 10.1016/j.phytochem.2021.112713 33667813

[B8] Gong Q. Hu J. Wang P. Li X. Zhang X. (2021). A comprehensive review on β-lapachone: mechanisms, structural modifications, and therapeutic potentials. Eur. J. Med. Chem. 210, 112962. 10.1016/j.ejmech.2020.112962 33158575

[B9] Gu Z. Xia J. Xu H. Frech I. Tricot G. Zhan F. (2017). NEK2 promotes aerobic glycolysis in multiple myeloma through regulating splicing of pyruvate kinase. J. Hematol. Oncol. 10, 17. 10.1186/s13045-017-0392-4 28086949 PMC5237262

[B10] Hisano Y. Hla T. (2019). Bioactive lysolipids in cancer and angiogenesis. Pharmacol. Ther. 193, 91–98. 10.1016/j.pharmthera.2018.07.006 30048709 PMC6309747

[B11] Hong S. M. Hwang S. W. Wang T. Park C. W. Ryu Y. M. Jung J. H. (2019). Increased nicotinamide adenine dinucleotide pool promotes colon cancer progression by suppressing reactive oxygen species level. Cancer Sci. 110, 629–638. 10.1111/cas.13886 30457689 PMC6361564

[B12] Huang X. Hou Y. Weng X. Pang W. Hou L. Liang Y. (2021). Diethyldithiocarbamate-copper complex (CuET) inhibits colorectal cancer progression via miR-16-5p and 15b-5p/ALDH1A3/PKM2 axis-mediated aerobic glycolysis pathway. Oncogenesis 10, 4. 10.1038/s41389-020-00295-7 33419984 PMC7794448

[B13] Huang X. Motea E. A. Moore Z. R. Yao J. Dong Y. Chakrabarti G. (2016). Leveraging an NQO1 bioactivatable drug for tumor-selective use of poly(ADP-ribose) polymerase inhibitors. Cancer Cell 30, 940–952. 10.1016/j.ccell.2016.11.006 27960087 PMC5161231

[B14] Kee J. Y. Han Y. H. Park J. Kim D. S. Mun J. G. Ahn K. S. (2017). β-Lapachone inhibits lung metastasis of colorectal cancer by inducing apoptosis of CT26 cells. Integr. Cancer Ther. 16, 585–596. 10.1177/1534735416681638 27923905 PMC5739146

[B15] Kitamura K. Hatano E. Higashi T. Narita M. Seo S. Nakamoto Y. (2011). Proliferative activity in hepatocellular carcinoma is closely correlated with glucose metabolism but not angiogenesis. J. Hepatol. 55, 846–857. 10.1016/j.jhep.2011.01.038 21334407

[B16] Lamberti M. J. Morales Vasconsuelo A. B. Chiaramello M. Ferreira V. F. Macedo Oliveira M. Baptista Ferreira S. (2018). NQO1 induction mediated by photodynamic therapy synergizes with β-Lapachone-halogenated derivative against melanoma. Biomed. Pharmacother. 108, 1553–1564. 10.1016/j.biopha.2018.09.159 30372857

[B17] Li L. S. Bey E. A. Dong Y. Meng J. Patra B. Yan J. (2011). Modulating endogenous NQO1 levels identifies key regulatory mechanisms of action of β-lapachone for pancreatic cancer therapy. Clin. Cancer Res. 17, 275–285. 10.1158/1078-0432.CCR-10-1983 21224367 PMC4806682

[B18] Li S. Cong X. Gao H. Lan X. Li Z. Wang W. (2019). Tumor-associated neutrophils induce EMT by IL-17a to promote migration and invasion in gastric cancer cells. J. Exp. Clin. Cancer Res. 38, 6. 10.1186/s13046-018-1003-0 30616627 PMC6323742

[B19] Liu F. Ma F. Wang Y. Hao L. Zeng H. Jia C. (2017). PKM2 methylation by CARM1 activates aerobic glycolysis to promote tumorigenesis. Nat. Cell Biol. 19, 1358–1370. 10.1038/ncb3630 29058718 PMC5683091

[B20] Lugano R. Ramachandran M. Dimberg A. (2020). Tumor angiogenesis: causes, consequences, challenges and opportunities. Cell Mol. Life Sci. 77, 1745–1770. 10.1007/s00018-019-03351-7 31690961 PMC7190605

[B21] Mahar R. Chang M. C. Merritt M. E. (2021). Measuring NQO1 bioactivation using [2H7]Glucose. Cancers (Basel) 13, 4165. 10.3390/cancers13164165 34439319 PMC8392257

[B22] Plate K. H. Scholz A. Dumont D. J. (2012). Tumor angiogenesis and anti-angiogenic therapy in malignant gliomas revisited. Acta Neuropathol. 124, 763–775. 10.1007/s00401-012-1066-5 23143192 PMC3508273

[B23] Qadir M. I. Iqbal M. S. Khan R. (2022). β-Lapachone: a promising anticancer agent with a unique NQO1 specific apoptosis in pancreatic cancer. Curr. Cancer Drug Targets 22, 537–540. 10.2174/1568009622666220427121127 35490325

[B24] Reddy D. Ghosh P. Kumavath R. (2020). Strophanthidin attenuates MAPK, PI3K/AKT/mTOR, and wnt/β-catenin signaling pathways in human cancers. Front. Oncol. 9, 1469. 10.3389/fonc.2019.01469 32010609 PMC6978703

[B25] Ross D. Siegel D. (2017). Functions of NQO1 in cellular protection and CoQ10 metabolism and its potential role as a redox sensitive molecular switch. Front. Physiol. 8, 595. 10.3389/fphys.2017.00595 28883796 PMC5573868

[B26] Sanajou D. Bahrambeigi S. Aslani S. (2021). β-LAPachone is renoprotective in streptozotocin-induced diabetic mice via regulating the PI3K/Akt/mTOR signaling pathway. Iran. J. Basic Med. Sci. 24, 650–656. 10.22038/ijbms.2021.55565.12422 34249267 PMC8244603

[B27] Sharma V. R. Gupta G. K. Sharma A. K. Batra N. Sharma D. K. Joshi A. (2017). PI3K/Akt/mTOR intracellular pathway and breast cancer: factors, mechanism and regulation. Curr. Pharm. Des. 23, 1633–1638. 10.2174/1381612823666161116125218 27848885

[B28] Shibuya M. (2011). Vascular endothelial growth factor (VEGF) and its receptor (VEGFR) signaling in angiogenesis: a crucial target for anti- and pro-angiogenic therapies. Genes Cancer 2, 1097–1105. 10.1177/1947601911423031 22866201 PMC3411125

[B29] Siegel R. L. Giaquinto A. N. Jemal A. (2024). Cancer statistics, 2024. CA Cancer J. Clin. 74 (1), 12–49. 10.3322/caac.21820 38230766

[B30] Silvers M. A. Deja S. Singh N. Egnatchik R. A. Sudderth J. Luo X. (2017). The NQO1 bioactivatable drug, β-lapachone, alters the redox state of NQO1+ pancreatic cancer cells, causing perturbation in central carbon metabolism. J. Biol. Chem. 292, 18203–18216. 10.1074/jbc.M117.813923 28916726 PMC5672043

[B31] Singh M. Yelle N. Venugopal C. Singh S. K. (2018). EMT: mechanisms and therapeutic implications. Pharmacol. Ther. 182, 80–94. 10.1016/j.pharmthera.2017.08.009 28834698

[B32] Sun Q. Chen X. Ma J. Peng H. Wang F. Zha X. (2011). Mammalian target of rapamycin up-regulation of pyruvate kinase isoenzyme type M2 is critical for aerobic glycolysis and tumor growth. Proc. Natl. Acad. Sci. U. S. A. 108, 4129–4134. 10.1073/pnas.1014769108 21325052 PMC3054028

[B33] Tamada M. Suematsu M. Saya H. (2012). Pyruvate kinase M2: multiple faces for conferring benefits on cancer cells. Clin. Cancer Res. 18, 5554–5561. 10.1158/1078-0432.CCR-12-0859 23071357

[B34] Tang Z. Li C. Kang B. Gao G. Li C. Zhang Z. (2017). GEPIA: a web server for cancer and normal gene expression profiling and interactive analyses. Nucleic Acids Res. 45 (W1), W98–W102. 10.1093/nar/gkx247 28407145 PMC5570223

[B35] Vaccarella S. Lortet-Tieulent J. Plummer M. Franceschi S. Bray F. (2013). Worldwide trends in cervical cancer incidence: impact of screening against changes in disease risk factors. Eur. J. Cancer 49, 3262–3273. 10.1016/j.ejca.2013.04.024 23751569

[B36] Woo H. Y. Kim H. S. (2022). Local and metastatic relapses in a young woman with papillary squamous cell carcinoma of the uterine cervix. Diagn. (Basel) 12, 599. 10.3390/diagnostics12030599 PMC8946994 35328152

[B37] Wu H. Yang P. Hu W. Wang Y. Lu Y. Zhang L. (2016). Overexpression of PKM2 promotes mitochondrial fusion through attenuated p53 stability. Oncotarget 7, 78069–78082. 10.18632/oncotarget.12942 27801666 PMC5363644

[B38] Yang J. Nie J. Ma X. Wei Y. Peng Y. Wei X. (2019). Targeting PI3K in cancer: mechanisms and advances in clinical trials. Mol. Cancer 18, 26. 10.1186/s12943-019-0954-x 30782187 PMC6379961

[B39] Yang Y. Zhou X. Xu M. Piao J. Zhang Y. Lin Z. (2017). β-lapachone suppresses tumour progression by inhibiting epithelial-to-mesenchymal transition in NQO1-positive breast cancers. Sci. Rep. 7, 2681. 10.1038/s41598-017-02937-0 28578385 PMC5457413

[B40] Yang Y. C. Cheng T. Y. Huang S. M. Su C. Y. Yang P. W. Lee J. M. (2016). Cytosolic PKM2 stabilizes mutant EGFR protein expression through regulating HSP90-EGFR association. Oncogene 35, 3387–3398. 10.1038/onc.2015.397 26500058

[B41] Yu H. Y. Kim S. O. Jin C. Y. Kim G. Y. Kim W. J. Yoo Y. H. (2014). β-lapachone-Induced apoptosis of human gastric carcinoma AGS cells is caspase-dependent and regulated by the PI3K/akt pathway. Biomol. Ther. Seoul. 22, 184–192. 10.4062/biomolther.2014.026 25009698 PMC4060078

[B42] Zhao W. Jiang L. Fang T. Fang F. Liu Y. Zhao Y. (2021). β-Lapachone selectively kills hepatocellular carcinoma cells by targeting NQO1 to induce extensive DNA damage and PARP1 hyperactivation. Front. Oncol. 11, 747282. 10.3389/fonc.2021.747282 34676172 PMC8523939

[B43] Zheng Y. Xie L. Xu S. Yan W. Zhang H. Meng Y. (2021b). Effects of miR-202-5p silencing PIK3CA gene expression on proliferation, invasion, and epithelial-mesenchymal transition of cervical cancer SiHa cells through inhibiting PI3K/Akt/mTOR signaling pathway activation. Mol. Cell Biochem. 476, 4031–4044. 10.1007/s11010-021-04211-4 34244973

[B44] Zheng Y. Zhang H. Guo Y. Chen Y. Chen H. Liu Y. (2021a). X-ray repair cross-complementing protein 1 (XRCC1) loss promotes β-lapachone -induced apoptosis in pancreatic cancer cells. BMC Cancer 21, 1234. 10.1186/s12885-021-08979-y 34789190 PMC8600733

[B45] Zhou Y. Yan H. Zhou Q. Feng R. Wang P. Yang F. (2021). Beta-lapachone attenuates BMSC-mediated neuroblastoma malignant transformation by inhibiting gal-3/gal-3BP/IL6 Axis. Front. Pharmacol. 12, 766909. 10.3389/fphar.2021.766909 34790130 PMC8591123

[B46] Zhu K. Deng C. Du P. Liu T. Piao J. Piao Y. (2022). G6PC indicated poor prognosis in cervical cancer and promoted cervical carcinogenesis *in vitro* and *in vivo* . Reprod. Biol. Endocrinol. 20, 50. 10.1186/s12958-022-00921-6 35277194 PMC8915493

[B47] Zhu P. Lu J. Zhi X. Zhou Y. Wang X. Wang C. (2021). tRNA-derived fragment tRFLys-CTT-010 promotes triple-negative breast cancer progression by regulating glucose metabolism via G6PC. Carcinogenesis 42, 1196–1207. 10.1093/carcin/bgab058 34216208

