# Supplementary material for: β-lapachone suppresses carcinogenesis of cervical cancer via interaction with AKT1
Source: Front Pharmacol. 2025 Feb 20;16:1509568. doi: 10.3389/fphar.2025.1509568 (PMC11882534; doi:10.3389/fphar.2025.1509568)
Supplement: Supplementary file 1 [file DataSheet1.docx]

**Supplementary Figures:**

**Fig.S1.** After treating human normal cervical and cancerous epithelial cells with various concentrations of β-lapachone, MTT assays were conducted to assess cell viability.


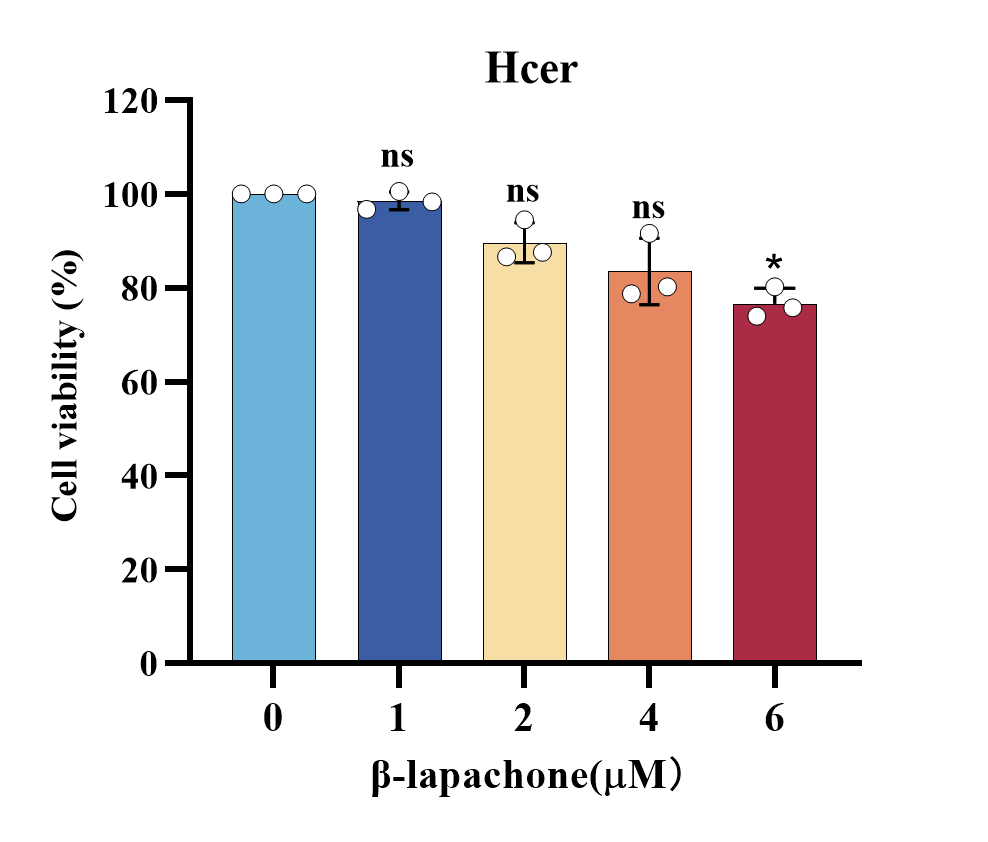

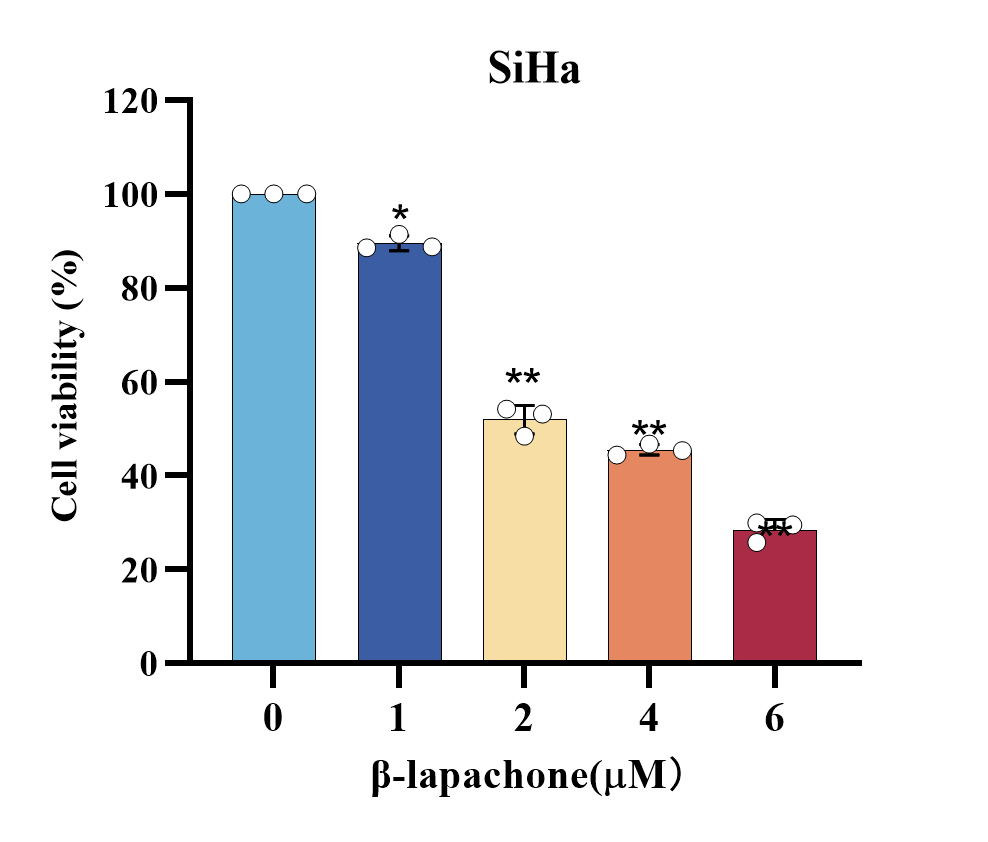

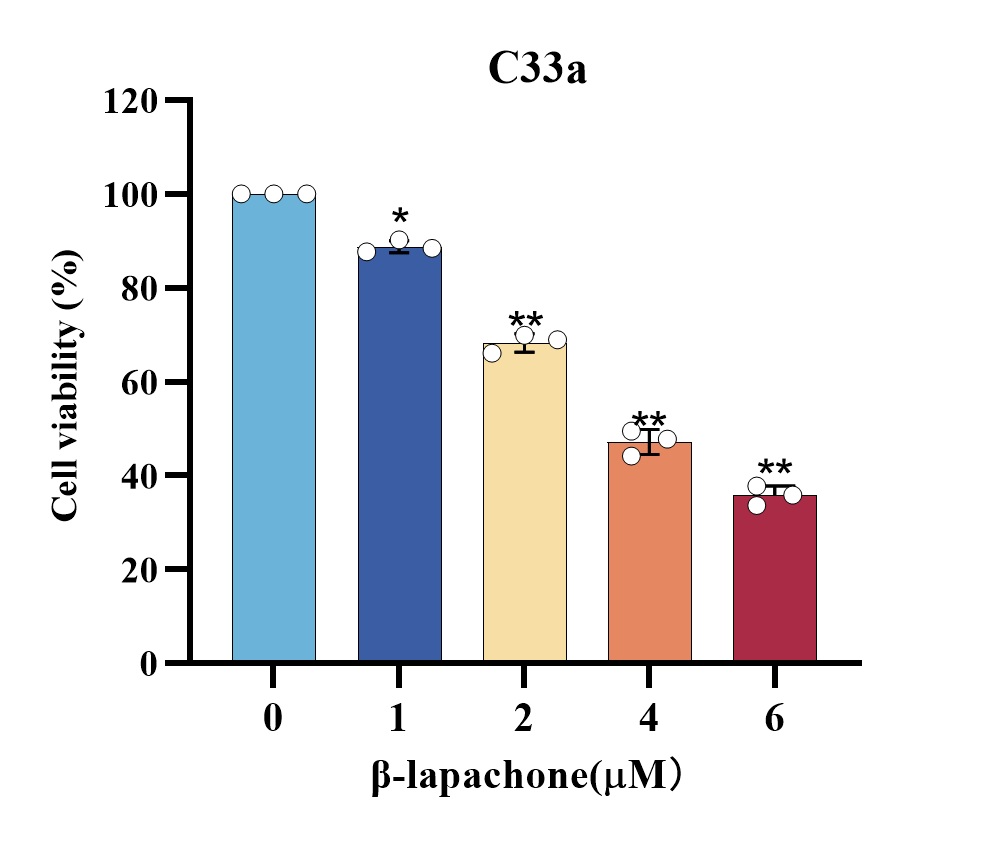


Error bars represent the means ± SD of three independent experiments. ns: *P* > 0.05, **P* < 0.05, ***P* < 0.01 vs 0 μM β-lapachone group.
